# Supplementary material for: Naked mole-rat very-high-molecular-mass hyaluronan exhibits superior cytoprotective properties
Source: Nat Commun. 2020 May 12;11:2376. doi: 10.1038/s41467-020-16050-w (PMC7217962; doi:10.1038/s41467-020-16050-w)
Supplement: Supplementary file 1 — Supplementary Information [file 41467_2020_16050_MOESM1_ESM.pdf]

Supplementary Information

Naked mole-rat very-high-molecular-mass hyaluronan exhibits superior cytoprotective properties

Masaki Takasugi, Denis Firsanov, Gregory Tombline, Hanbing Ning, Julia Ablaeva, Andrei Seluanov, Vera Gorbunova

Correspondence to: vera.gorbunova@rochester.edu and andrei.seluanov@rochester.edu

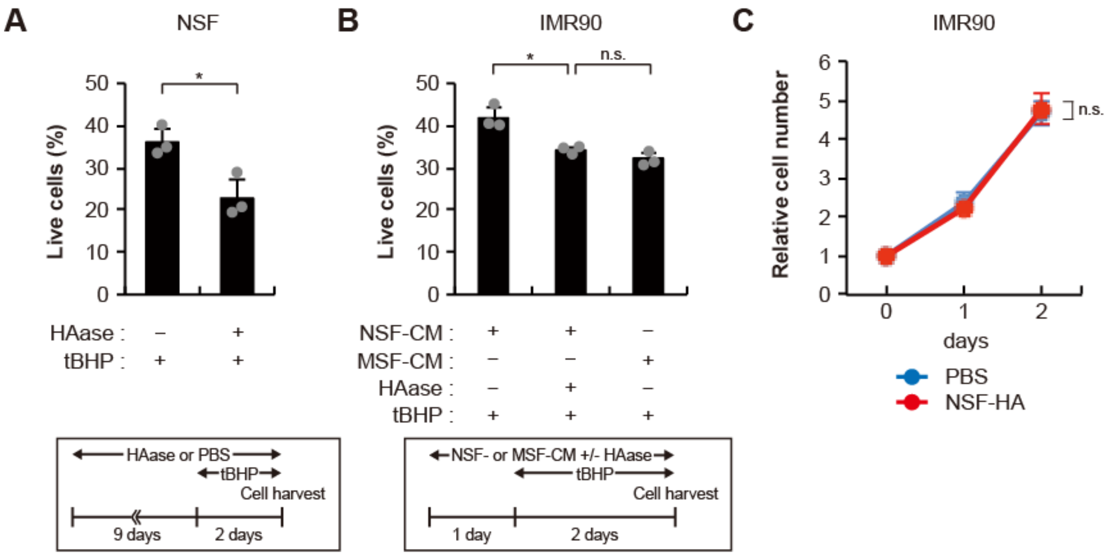

Supplementary Figure 1 | The effects of NSF-HA on cell death and cell proliferation. (**A**) Cell survival of NSF cells upon oxidative stress is dependent on NSF-HA. The percentages of live cells were measured by Annexin-V/PI staining after 2 days of 200 µM tBHP-treatment (n = 3). Culture media were supplemented with HAase or PBS from 9 days before tBHP-treatment until the end of the experiment. HAase (3 U/ml) or PBS was added to the media on 9, 6, and 3 days before tBHP-treatment. Then the media were replaced with fresh media containing tBHP and HAase or PBS. (**B**) NSF conditioned media enhance cell survival of IMR90 cells upon oxidative stress compared to that of MSF in a NSF-HA-dependent manner. The percentages of live cells were measured by Annexin-V/PI staining after 2 days of 40 µM tBHP-treatment (n = 3). Cells were cultured in NSF or MSF conditioned media with or without HAase (3U/ml) from 1 day before tBHP-treatment until the end of the experiment. (**C**) NSF-HA has no effect on proliferation of IMR90 cells in the absence of oxidative stress. The plot shows the relative numbers of IMR90 cells cultured in the media containing 20 µg/ml NSF-HA or PBS (n = 4). The media were changed every day. Error bars are presented as mean ± SD values. * $p < 0.05$ [two-tailed t-test for (**A, C**) and one-way ANOVA with *post-hoc* Dunnett's two-tailed test for (**B**)].

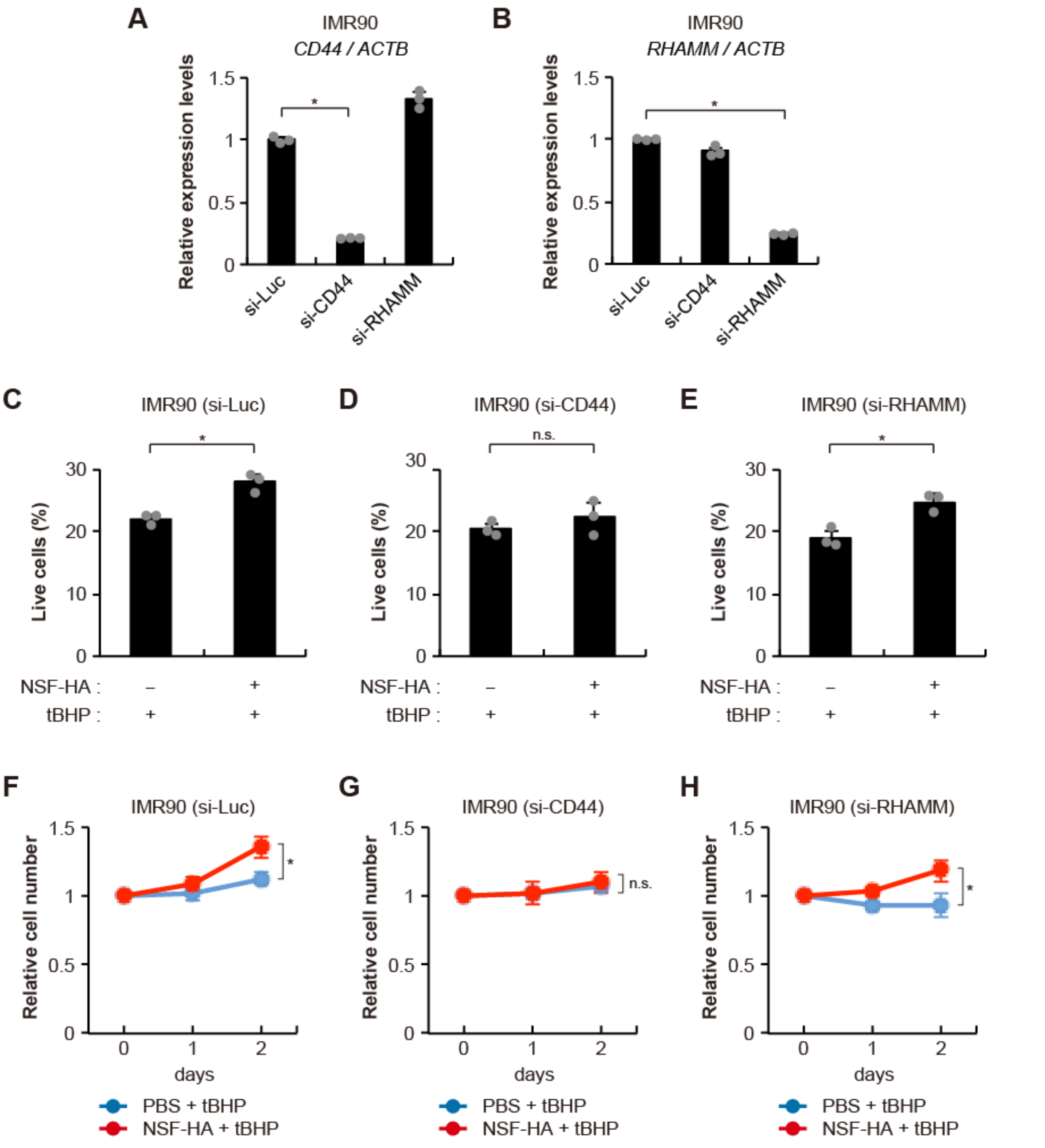

Supplementary Figure 2 | The cytoprotective effect of NSF-HA depends on CD44. (**A, B**) RT-qPCR analyses of CD44 and RHAMM in IMR90 cells (*n* = 3). ACTB was used as an internal control. Cells were transfected with indicated siRNAs 2 days before sample collection. (**C-E**) CD44-, but not RHAMM-knockdown abrogates the cytoprotective effect of NSF-HA on oxidative stress-induced cell death in IMR90 cells. The percentages of live cells were measured by Annexin-V/PI staining 1 day after 1 h of 3 mM tBHP-treatment (*n* = 3). Cells were pre-incubated for 6 h with 20 μg/ml NSF-HA or PBS before tBHP-treatment. siRNAs were

transfected 2 days before the first tBHP-treatment. (**F-H**) CD44-, but not RHAMM-knockdown abrogates the cytoprotective effect of NSF-HA on oxidative stress-induced growth arrest in IMR90 cells (n = 4). Cells were pre-incubated for 6 h with 20 μg/ml NSF-HA or PBS and then HA was removed and cells were exposed to 200 μM tBHP for 1 h on day 0 and day 1. siRNAs were transfected 2 days before day 0. Error bars are presented as mean ± SD values. * $p < 0.05$ [one-way ANOVA with *post-hoc* Dunnett's two-tailed test for (**A, B**) and two-tailed t-test for (**C-H**)].

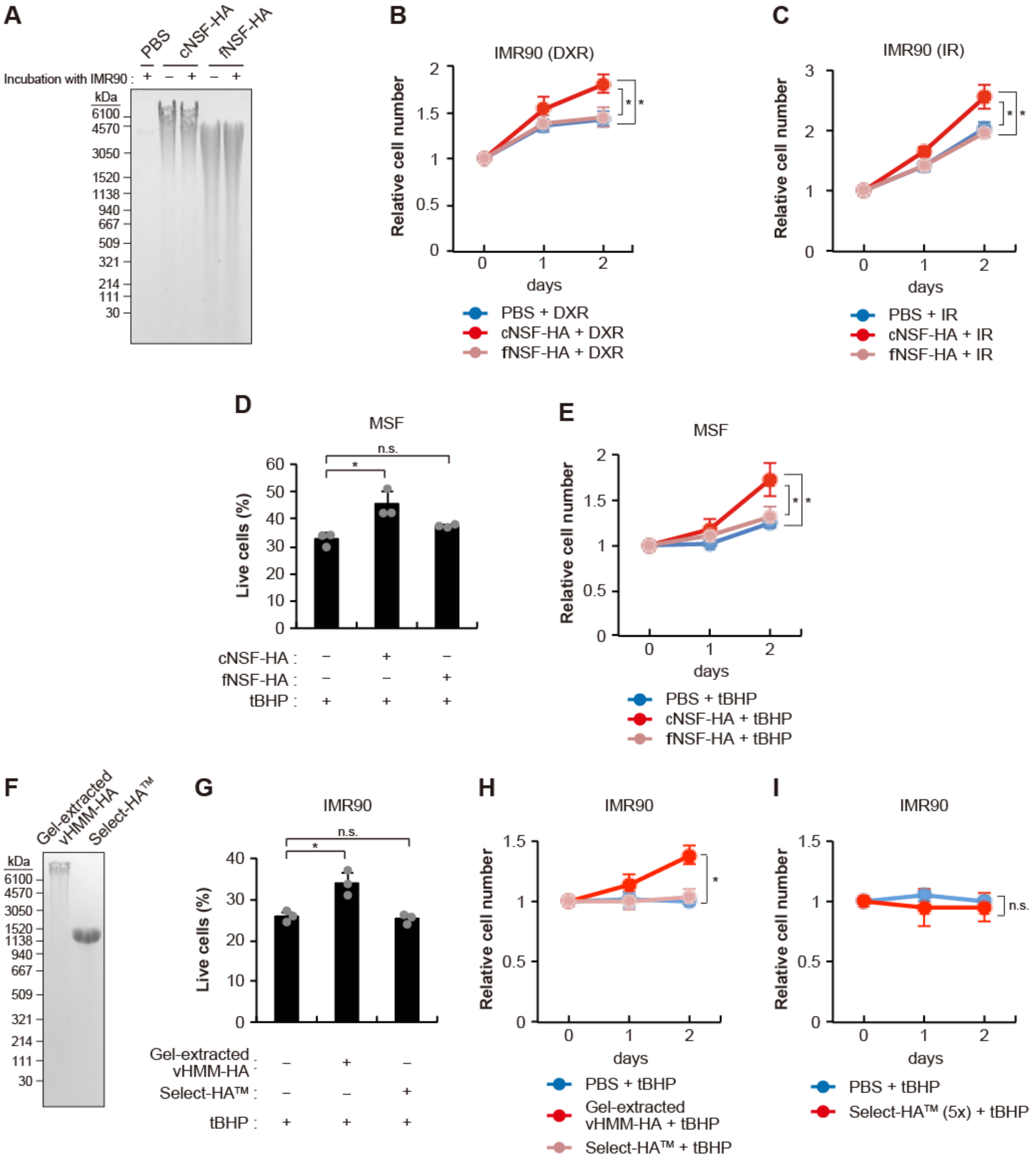

Supplementary Figure 3 | The cytoprotective effect of NSF-HA depends on HA polymer length. (**A**) HA length does not change during incubation with IMR90 cells. Pulse-field gel electrophoresis image of cNSF-HA and fNSF-HA. HA was added to the media at the concentration of 20 μg/ml and incubated with or without IMR90 cells and purified again after 6 h of incubation. The experiment was repeated once with similar result. (**B, C**) cNSF-HA, but not

fNSF-HA, suppresses growth arrest induced by various stresses in IMR90 cells (n = 4). Cells were incubated for 6 h with 20 μg/ml cNSF-HA, fNSF-HA, or PBS on day 0 before exposing cells to stresses and on day 1. After the pre-incubation on day 0, cells were exposed to 400 ng/ml doxorubicin (DXR) **(B)** or irradiated with 3 Gy gamma irradiation **(C)**. (**D**) cNSF-HA, but not fNSF-HA, suppresses oxidative stress-induced cell death in MSF. The percentages of live MSF were measured by Annexin-V/PI staining 1 day after 1 h of 1.5 mM tBHP-treatment (n = 3). Cells were pre-incubated for 6 h with 20 μg/ml cNSF-HA, fNSF-HA, or PBS before tBHP-treatment. (**E**) cNSF-HA, but not fNSF-HA, suppresses oxidative stress-induced growth arrest in MSF (n = 4). Cells were pre-incubated for 6 h with 20 μg/ml cNSF-HA, fNSF-HA, or PBS and then HA was removed and cells were exposed to 150 μM tBHP for 1 h on day 0 and day 1. (**F**) Size comparison of gel-extracted vHMM-HA and Select-HA™. Pulse-field gel electrophoresis image of gel-extracted vHMM-HA and Select-HA™ is shown. The experiment was repeated once with similar result. (**G**) Gel-extracted vHMM-HA, but not Select-HA™, suppresses oxidative stress-induced cell death in IMR90 cells. The percentages of live cells were measured by Annexin-V/PI staining 1 day after 1 h of 3 mM tBHP-treatment (n = 3). Cells were pre-incubated for 6 h with 20 μg/ml gel-extracted vHMM-HA, Select-HA™, or PBS before tBHP-treatment. (**H**) Gel-extracted vHMM-HA, but not Select-HA™, suppresses oxidative stress-induced growth arrest in IMR90 cells (n = 4). Cells were pre-incubated for 6 h with 20 μg/ml ge-extracted vHMM-HA, Select-HA™, or PBS and then HA was removed and cells were exposed to 200 μM tBHP for 1 h on day 0 and day 1. (**I**) Select-HA™ does not suppress oxidative stress-induced growth arrest in IMR90 cells even at the concentration of 100 μg/ml (n = 4). Cells were pre-incubated for 6 h with 100 μg/ml Select-HA™ or PBS and then HA was removed and cells were exposed to 200 μM tBHP for 1 h on day 0 and day 1. Error bars are presented as mean ± SD values. * $p < 0.05$ [one-way ANOVA with *post-hoc* Dunnett's two-tailed test for (**B, C, D, E, G, H**) and two-tailed t-test for (**I**)].

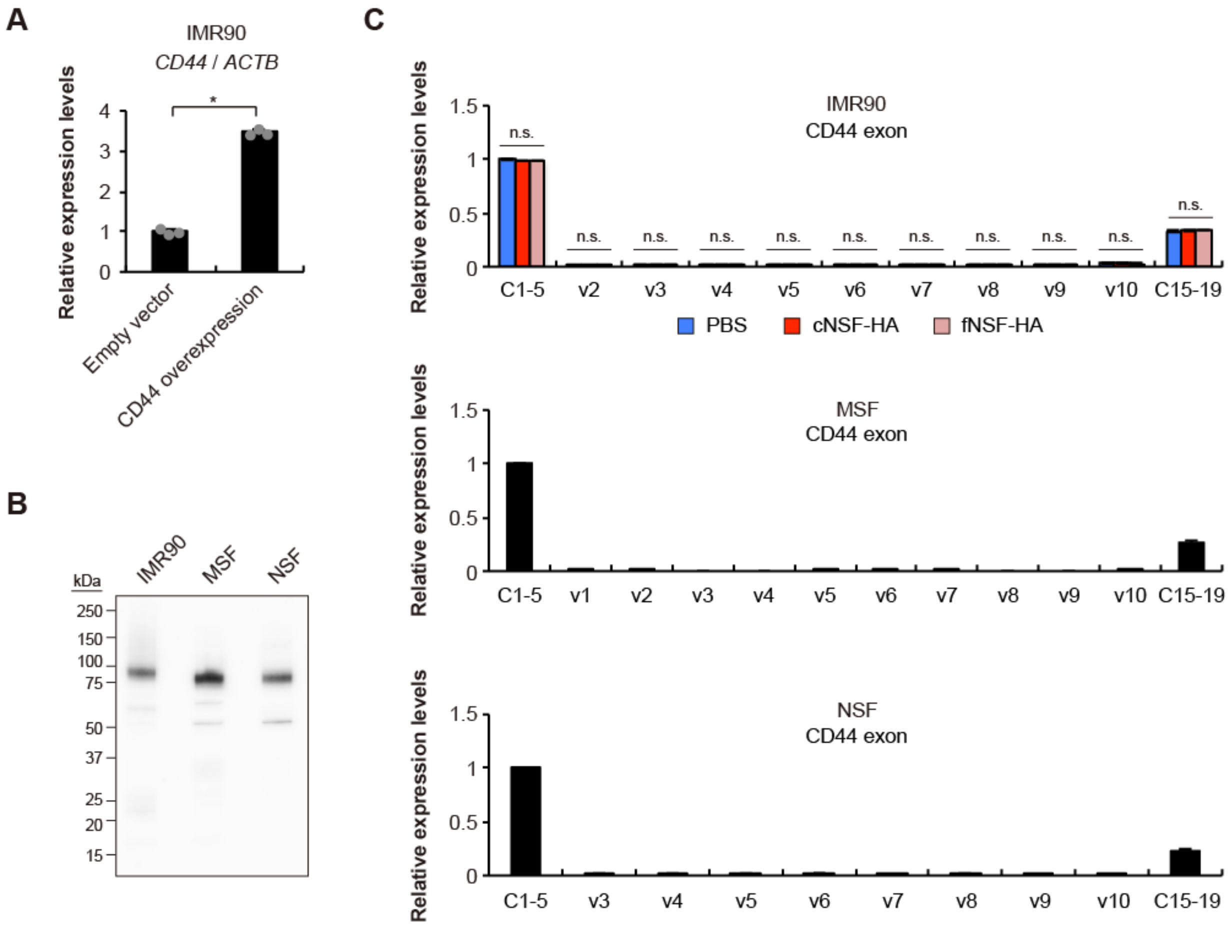

Supplementary Figure 4 | Expression analyses of CD44. (**A**) Relative expression levels (TPM) of CD44 in CD44-overexpressing IMR90 cells (*n* = 3). (**B**) Western blot showing the expression of CD44 in IMR90 cells, MSF, and NSF. The experiment was repeated once with similar result. (**C**) Relative expression levels (TPM) of constant and variant CD44 exons in IMR90 cells, MSF, and NSF (*n* = 3). RNA-Seq data collected in our current and previous studies were used. Error bars are presented as mean ± SD values. * $p < 0.05$ [two-tailed t-test for (**A**) and one-way ANOVA with *post-hoc* Dunnett's two-tailed test for (**C**)].

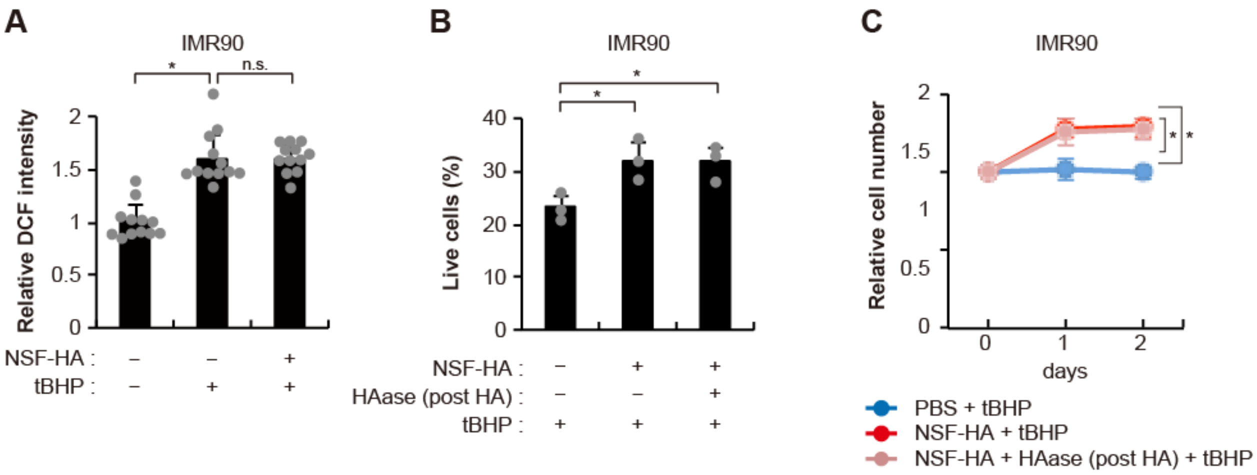

Supplementary Figure 5 | The effects of NSF-HA on ROS levels. (**A**) NSF-HA does not affect tBHP-induced increase in intracellular ROS levels. Intracellular ROS levels were measured by DCF fluorescence in IMR90 cells (*n* = 12). Cells were pre-incubated for 6 h with 20 μg/ml NSF-HA or PBS and then HA was removed and cells were exposed to 200 μM tBHP for 1 h. Assay was performed right after the tBHP-treatment. (**B, C**) Digestion of residual HA after the 6 h NSF-HA incubation does not affect the cytoprotective effect. (**B**) The percentages of live cells were measured by Annexin-V/PI staining 1 day after 1 h of 3 mM tBHP-treatment (n = 3). Cells were pre-incubated for 6 h with 20 μg/ml NSF-HA or PBS and then with 10 U/ml HAase or PBS for 30 min before tBHP-treatment. (**C**) The plot shows the relative numbers of IMR90 cells (*n* = 4). Cells were pre-incubated for 6 h with 20 μg/ml NSF-HA or PBS and then with 10 U/ml HAase for 30 min, and then exposed to 200 μM tBHP for 1 h on day 0 and day 1. Error bars are presented as mean ± SD values. * $p < 0.05$ (one-way ANOVA with *post-hoc* Dunnett's two-tailed test).

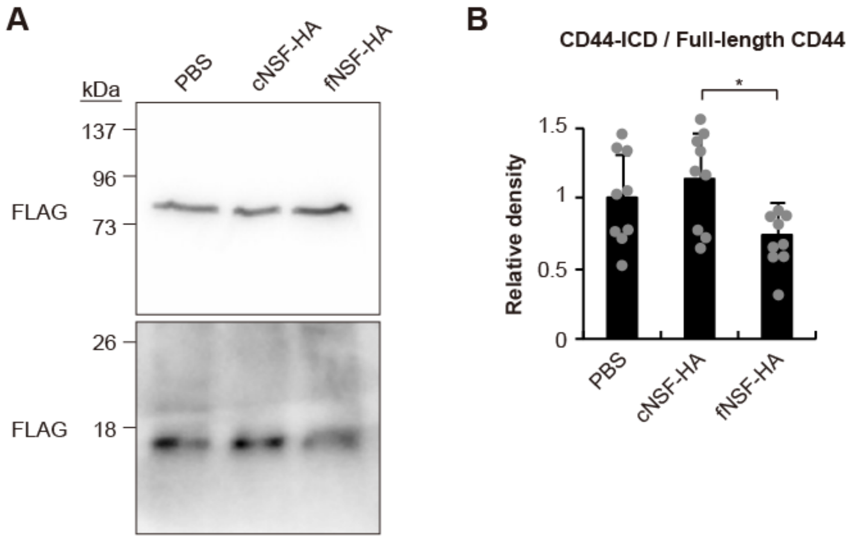

Supplementary Figure 6 | The effects of cNSF-HA and fNSF-HA on CD44-ICD levels. (**A**) Western blots showing full-length CD44-FLAG and CD44-ICD-FLAG levels in CD44-FLAG overexpressing IMR90 cells. Cells were incubated for 6 h with 20 μg/ml cNSF-HA, fNSF-HA or PBS. (**B**) Quantification of Western blots shown in (A) (n = 9). Error bars are presented as mean ± SD values. * $p < 0.05$ (one-way ANOVA with *post-hoc* Dunnett's two-tailed test).

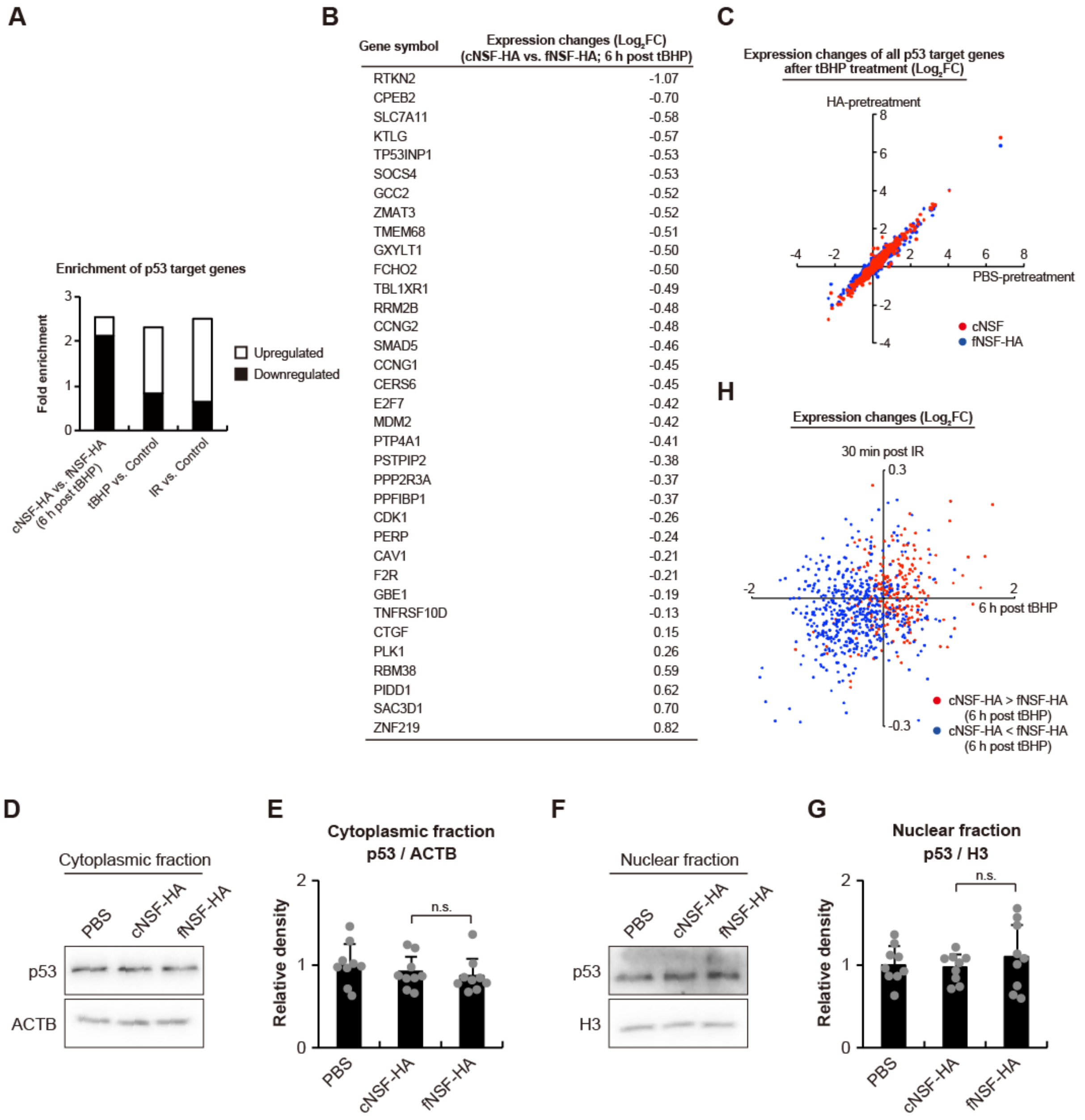

Supplementary Figure 7 | cNSF-HA suppresses a subset of p53 target gene expressions. (**A**) Fold enrichment of p53 target genes in the HA polymer length-dependent genes and in the genes affected by 1 h of 200 µM tBHP-treatment and 5 Gy irradiation. Expression analyses were conducted 6 h and 30 min after the tBHP-treatment and irradiation, respectively. (**B**) Differential expression levels of the HA polymer length-dependent p53 target genes in cNSF-HA- and fNSF-HA pre-incubated IMR90 cells. Cells were pre-incubated for 6 h with 20 µg/ml cNSF-HA or fNSF-HA and then HA was removed and cells were exposed to 200 µM tBHP for 1 h. Cells were collected 6 h after starting the tBHP-treatment. (**C**) Expression changes of all p53 target

genes induced in IMR90 cells by 1 h of 200 μM tBHP-treatment. Expression changes induced in PBS pre-incubated IMR90 cells are plotted against expression changes induced in HA pre-incubated IMR90 cells. Cells were pre-incubated for 6 h with 20 μg/ml cNSF-HA, fNSF-HA, or PBS and then HA was removed and cells were exposed to 200 μM tBHP for 1 h. (**D, F**) Western blots showing p53 levels in cytoplasmic and nuclear fractions of IMR90 cells. Cells were incubated for 6 h with 20 μg/ml cNSF-HA, fNSF-HA, or PBS (n = 9). (**E, G**) Quantification of Western blots shown in (D, F) by Image J. (**H**) Expression changes induced in IMR90 cells by 1 h of 200 μM tBHP-treatment and 5 Gy irradiation are plotted for the HA polymer length-dependent genes. Expression analyses were conducted 6 h and 30 min after the tBHP-treatment and irradiation, respectively. Red and blue dots represent the genes that are up- and downregulated in cNSF-HA pre-incubated cells, respectively. Error bars are presented as mean ± SD values. n.s. $p \geqq 0.05$ (one-way ANOVA with *post-hoc* Dunnett's two-tailed test).

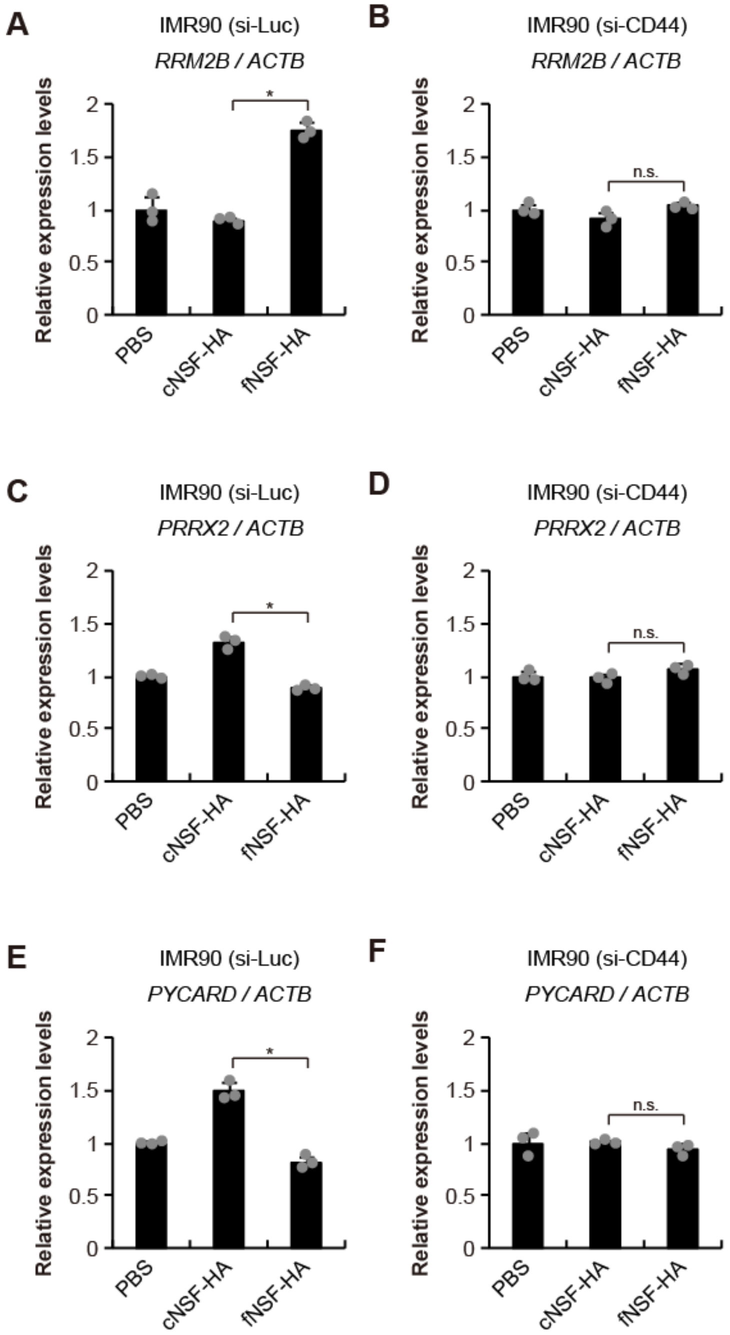

Supplementary Figure 8 | The effects of HA on the expression levels of p53 target genes depend on CD44. (**A-F**) RT-qPCR analyses of RRM2B, PRRX2, and PYCARD in IMR90 cells ($n = 3$). Cells were incubated for 6 h with 20 μg/ml cNSF-HA, fNSF-HA, or PBS and were transfected with indicated siRNAs 2 days before sample collection. Error bars are presented as mean ± SD values. * $p < 0.05$ (one-way ANOVA with *post-hoc* Dunnett's two-tailed test).

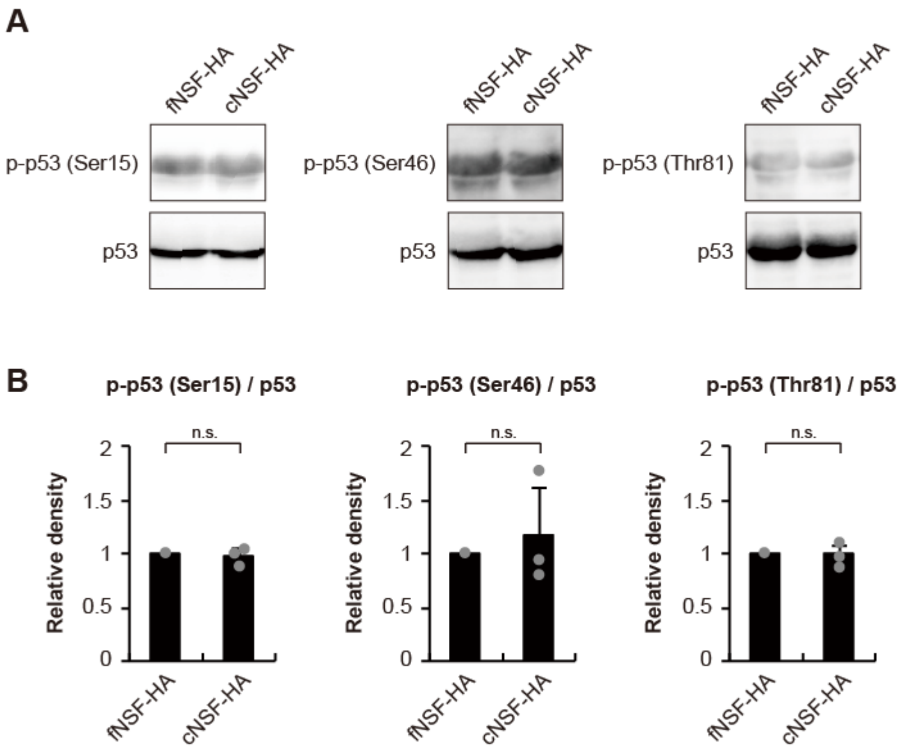

Supplementary Figure 9 | The effects of cNSF-HA and fNSF-HA on p53 phosphorylation. (**A**) Western blots showing p53 phosphorylation levels in IMR90 cells treated with cNSF-HA or fNSF-HA. Cells were incubated for 6 h with 20 μg/ml cNSF-HA or fNSF-HA. (**B**) Quantification of Western blots shown in (A) by Image J. Averages are of 3 biological replicates. For each biological replicate, immunoblotting was performed 3 times and averaged. Error bars are presented as mean ± SD values. n.s. $p \geqq 0.05$ (two-tailed t-test).

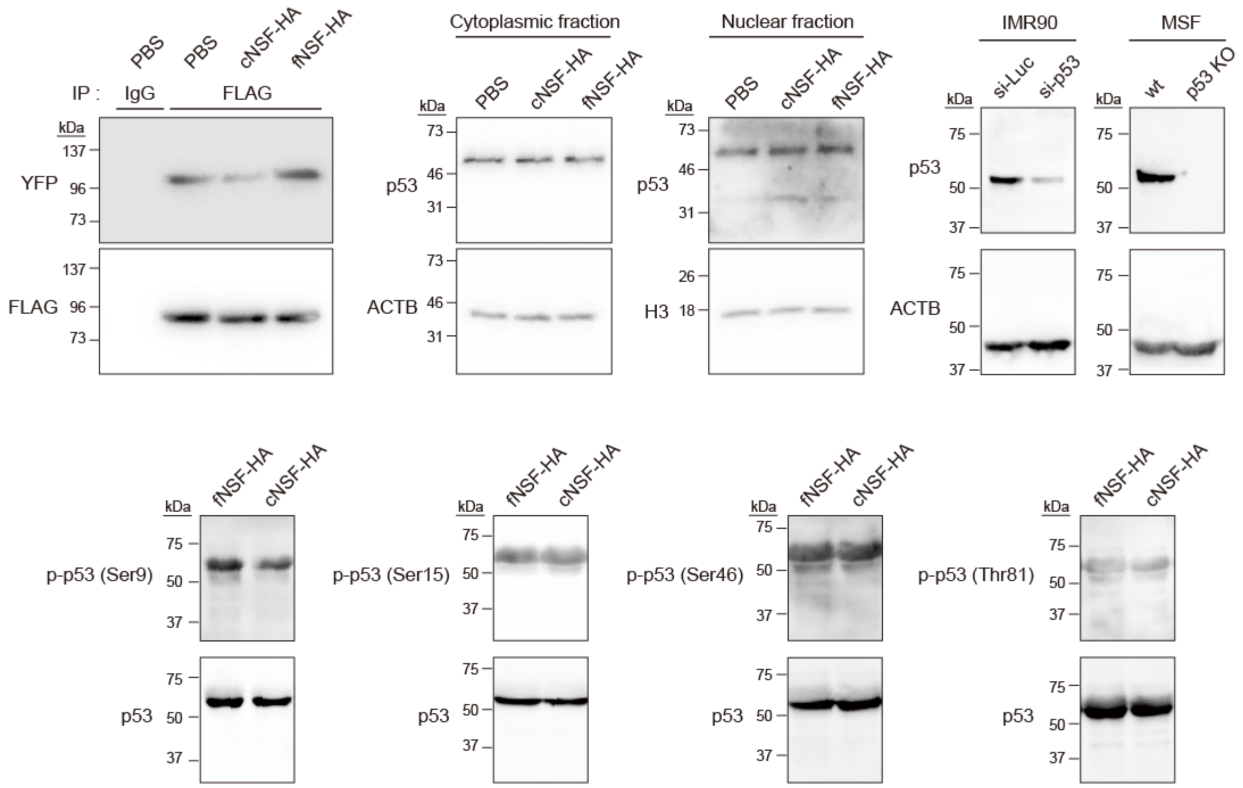

Supplementary Figure 10 | Uncropped gel images of western blots.
