# Supplementary material for: Naked mole-rat very-high-molecular-mass hyaluronan exhibits superior cytoprotective properties
Source: Nat Commun. 2020 May 12;11:2376. doi: 10.1038/s41467-020-16050-w (PMC7217962; doi:10.1038/s41467-020-16050-w)
Supplement: Supplementary file 4 — Reporting Summary [file 41467_2020_16050_MOESM4_ESM.pdf]

nature research

Corresponding author(s): Vera Gorbunova, Andrei Seluanov

Last updated by author(s): Mar 28, 2020

Reporting Summary

Nature Research wishes to improve the reproducibility of the work that we publish. This form provides structure for consistency and transparency in reporting. For further information on Nature Research policies, see Authors & Referees and the Editorial Policy Checklist.

Statistics

For all statistical analyses, confirm that the following items are present in the figure legend, table legend, main text, or Methods section.

n/a	Confirmed	
☐	☒	The exact sample size (n) for each experimental group/condition, given as a discrete number and unit of measurement
☐	☒	A statement on whether measurements were taken from distinct samples or whether the same sample was measured repeatedly
☐	☒	The statistical test(s) used AND whether they are one- or two-sided<br>*Only common tests should be described solely by name; describe more complex techniques in the Methods section.*
☐	☒	A description of all covariates tested
☒	☐	A description of any assumptions or corrections, such as tests of normality and adjustment for multiple comparisons
☐	☒	A full description of the statistical parameters including central tendency (e.g. means) or other basic estimates (e.g. regression coefficient) AND variation (e.g. standard deviation) or associated estimates of uncertainty (e.g. confidence intervals)
☐	☒	For null hypothesis testing, the test statistic (e.g. F, t, r) with confidence intervals, effect sizes, degrees of freedom and P value noted<br>*Give P values as exact values whenever suitable.*
☒	☐	For Bayesian analysis, information on the choice of priors and Markov chain Monte Carlo settings
☒	☐	For hierarchical and complex designs, identification of the appropriate level for tests and full reporting of outcomes
☒	☐	Estimates of effect sizes (e.g. Cohen's d, Pearson's r), indicating how they were calculated

Our web collection on statistics for biologists contains articles on many of the points above.

Software and code

Policy information about availability of computer code

Data collection	No software was used for data collection.
Data analysis	DeSeq2 ver 1.16.1, oPOSSUM 3.0, Enrichr, Image J 1.47v, Proteome Discoverer, Sequest

For manuscripts utilizing custom algorithms or software that are central to the research but not yet described in published literature, software must be made available to editors/reviewers. We strongly encourage code deposition in a community repository (e.g. GitHub). See the Nature Research guidelines for submitting code & software for further information.

Data

Policy information about availability of data

All manuscripts must include a data availability statement. This statement should provide the following information, where applicable:

- Accession codes, unique identifiers, or web links for publicly available datasets
- A list of figures that have associated raw data
- A description of any restrictions on data availability

Data availability. Source Data are provided as a Source Data file. RNA-Seq data have been deposited in the Gene Expression Omnibus (GEO) database under the accession number GSE116759 (https://www.ncbi.nlm.nih.gov/geo/query/acc.cgi?acc=GSE116759). Mass spectrometry data have been deposited in the ProteomeXchange with identifier PXD013445 (https://www.ebi.ac.uk/pride/archive/projects/PXD013445).

Field-specific reporting

Please select the one below that is the best fit for your research. If you are not sure, read the appropriate sections before making your selection.

☒ Life sciences ☐ Behavioural & social sciences ☐ Ecological, evolutionary & environmental sciences

For a reference copy of the document with all sections, see nature.com/documents/nr-reporting-summary-flat.pdf

Life sciences study design

All studies must disclose on these points even when the disclosure is negative.

Sample size	Sample size was set at >3 since we used student t-test or post-hoc Dunnet test to detect significant difference in most cases. Exact sample size used in this study was determined based on the expense of data collection.
Data exclusions	No data were excluded from the analysis.
Replication	All experiments were repeated at least 2 times to confirm the reproducibility of the results.
Randomization	Samples were allocated into experimental without any intention.
Blinding	Investigators were not blinded during the experiments, since this study did not involve human or animal experiment. We only used animals to establish cell lines and did not compare animals.

Reporting for specific materials, systems and methods

We require information from authors about some types of materials, experimental systems and methods used in many studies. Here, indicate whether each material, system or method listed is relevant to your study. If you are not sure if a list item applies to your research, read the appropriate section before selecting a response.

Materials & experimental systems

n/a	Involved in the study	
☐	☒	Antibodies
☐	☒	Eukaryotic cell lines
☒	☐	Palaeontology
☐	☒	Animals and other organisms
☒	☐	Human research participants
☒	☐	Clinical data

Methods

n/a	Involved in the study	
☒	☐	ChIP-seq
☒	☐	Flow cytometry
☒	☐	MRI-based neuroimaging

Antibodies

Antibodies used	anti-β-actin (sc-47778; Santa Cruz), anti-p53 (human) (10442-1-AP; Proteintech), anti-p53 (mouse) (ab26; Abcam), anti-phospho-p53 (Ser-9) (#9288; CST), anti-phospho-p53 (Ser-15) (#9286; CST), anti-phospho-p53 (Ser-46) (#2521; CST), anti-phospho-p53 (Thr-81) (#2676; CST), anti-CD44 (Clone 2C5; R&D), anti-CD44 (ab157107; Abcam), anti-CD44 (15675-1-AP; Proteintech), anti-γH2AX (JBW301; Millipore), anti-53BP1 (ab172580; Abcam), anti-FLAG (M2; sigma), anti-GFP (ab6556; abcam), anti-H3 (#9715; CST), anti-mouse IgG Alexa Fluor 488 (Thermo Fisher Scientific), anti-rabbit IgG Alexa Fluor 568 (Thermo Fisher Scientific)
Validation	anti-β-actin (sc-47778; Santa Cruz) : This antibody is validated for WB by the supplier (https://www.scbt.com/p/beta-actin-antibody-c4).<br>anti-p53 (human) (10442-1-AP; Proteintech) : This antibody is validated for WB by the supplier (https://www.ptglab.com/products/P53-Antibody-10442-1-AP.htm).<br>anti-p53 (mouse) (ab26; Abcam) : This antibody is validated for WB by the supplier (https://www.abcam.com/p53-antibody-pab-240-ab26.html.).<br>anti-phospho-p53 (Ser-9) (#9288; CST) : This antibody is validated for WB by the supplier (https://www.cellsignal.com/products/primary-antibodies/phospho-p53-ser9-antibody/9288).<br>anti-phospho-p53 (Ser-15) (#9286; CST) : This antibody is validated for WB by the supplier (https://www.cellsignal.com/products/primary-antibodies/phospho-p53-ser15-16g8-mouse-mab/9286).<br>anti-phospho-p53 (Ser-46) (#2521; CST) : This antibody is validated for WB by the supplier (https://en.cellsignal.jp/products/primary-antibodies/phospho-p53-ser46-antibody/2521).<br>anti-phospho-p53 (Thr-81) (#2676; CST) : This antibody is validated for WB by the supplier (https://en.cellsignal.jp/products/primary-antibodies/phospho-p53-thr81-antibody/2676).<br>anti-CD44 (Clone 2C5; R&D) : This antibody is validated for blocking CD44 in our previous work (Nature. 2013 Jul 18;499(7458):346-9. doi: 10.1038/nature12234.).<br>anti-CD44 (ab157107; Abcam) : This antibody is validated for WB and IP by the supplier (https://www.abcam.com/cd44-antibody-ab157107.html).

anti-CD44 (15675-1-AP; Proteintech) : This antibody is validated for WB and IP by the supplier (https://www.ptglab.com/products/CD44-Antibody-15675-1-AP.htm).
anti-γH2AX (JBW301; Millipore) : This antibody is validated for IF by the supplier (https://www.merckmillipore.com/JP/ja/product/Anti-phospho-Histone-H2A.X-Ser139-Antibody-clone-JBW301,MM_NF-05-636?ReferrerURL=https%3A%2F%2Fwww.google.com%2F#).
anti-53BP1 (ab172580; Abcam) : This antibody is validated for IF by the supplier (https://www.abcam.com/53bp1-antibody-ab172580.html).
anti-FLAG (M2; sigma) : This antibody is validated for WB and IP in previous works, such as Oncogene. 2017 Dec 14;36(50):6839-6849. doi: 10.1038/onc.2017.289.
anti-GFP (ab6556; abcam) : This antibody is validated for WB by the supplier (https://www.abcam.com/gfp-antibody-ab6556.html).
anti-H3 (#9715; CST) : This antibody is validated for WB by the supplier (https://en.cellsignal.jp/products/primary-antibodies/histone-h3-antibody/9715).
anti-mouse IgG Alexa Fluor 488 (Thermo Fisher Scientific) : This antibody is validated for IF by the supplier (https://www.thermofisher.com/antibody/product/Goat-anti-Mouse-IgG-H-L-Highly-Cross-Adsorbed-Secondary-Antibody-Polyclonal/A32723).
anti-rabbit IgG Alexa Fluor 568 (Thermo Fisher Scientific) : This antibody is validated for IF by the supplier (https://www.thermofisher.com/antibody/product/Goat-anti-Mouse-IgG-H-L-Highly-Cross-Adsorbed-Secondary-Antibody-Polyclonal/A-11031).

Eukaryotic cell lines

Policy information about cell lines

Cell line source(s)

Naked-mole-rat skin fibroblasts and mouse skin fibroblasts were isolated in our lab as described previously (Seluanov et al., J Vis Exp, 2010) from the skin of young adult NMRs and C57BL/6J mice, respectively. IMR90 human lung fibroblasts were from the Coriell Institute for Medical Research. HEK293T cells were from ATCC.

Authentication

Cell lines were authenticated based on unique morphology, growth characteristics, and hyaluronan synthesis activity of naked mole-rat, mouse, and human fibroblasts.

Mycoplasma contamination

We confirmed that IMR90 cells were mycoplasma-negative. Test was not performed for cells freshly isolated from animals in our lab.

Commonly misidentified lines
(See ICLAC register)

No commonly misidentified cell lines were used in the study

Animals and other organisms

Policy information about studies involving animals; ARRIVE guidelines recommended for reporting animal research

Laboratory animals

Adult male C57BL/6J mice and adult Naked mole-rats were used in this study

Wild animals

This study did not involve wild animals.

Field-collected samples

This study did not involve samples collected from the field.

Ethics oversight

Animals were maintained in accordance with the regulations designated and approved by the University of Rochester Committee on Animal Resources (UCAR), which adheres to FDA and NIH animal care guidelines and reviews all animal protocols prior to approval.

Note that full information on the approval of the study protocol must also be provided in the manuscript.
